# Genetic Modification for Agriculture—Proposed Revision of GMO Regulation in Australia

**DOI:** 10.3390/plants10040747

**Published:** 2021-04-11

**Authors:** Robert Redden

**Affiliations:** RJR Agriculture Consultants, 62 Schier Drive, Horsham 340, Australia; bbredden@yahoo.com.au

**Keywords:** genetic engineering, genome editing, regulation, climate change, precautionary principle

## Abstract

Genetic engineering (GM) of crops, modified with DNA transfer between species, has been highly regulated for over two decades. Now, genome editing (GE) enables a range of DNA alterations, from single base pair changes to precise gene insertion with site-directed nucleases (SDNs). Past regulations, established according to the precautionary principle of avoiding potential risks to human health and the environment, are predicated on fears fanned by well-funded and emotional anti-GM campaigns. These fears ignore the safety record of GM crops over the last 25 years and the benefits of GM to crop productivity, disease and pest resistance, and the environment. GE is now superseding GM, and public education is needed about its benefits and its potential to meet the challenges of climate change for crops. World population will exceed 9 billion by 2050, and world CO_2_ levels are now over 400 ppm in contrast with a pre-industrial 280 ppm, leading to a projected 1.5 °C global warming by 2050, with more stressful crop environments. The required abiotic and biotic stress tolerances can be introgressed from crop wild relatives (CWR) into domestic crops via GE. Restrictive regulations need to be lifted to facilitate GE technologies for sustainable agriculture in Australia and the world.

## 1. Introduction

GM crops in 2018 occupied 191.7 million hectares, providing improvement for the key traits of yield as well as disease and pest resistance across cereal, vegetable, oilseed and fiber crops [1,2,3]. Crops are genetically modified (GM) by inserting, removing, or altering the activity of one or more genes, or parts of a gene, so that an organism gains, loses or changes specific traits [4,5]. Previously, genetic modification involved a range of techniques, such as ballistics and site-directed DNA insertion using Talens, Zinc finger, and CRISPR (Clustered Regularly Interspaced Short Palindromic Repeats), with the latter now being predominant [6,7]. GM crops could not be bred with conventional plant breeding, as this involves the transfer of genes between non-crossable, or very difficult to cross, CWR and other species [8]. 

The development and release of GM crops is highly regulated and includes licensing, use of containment facilities for breeding, and approved isolation procedures for field trials [6,7,8,9,10]. The precautionary principle is applied to assess risks to health and to the environment. There are administrative requirements for approvals at each stage, including regulatory oversight, monitoring, and surveillance [4,11,12].

Revolutionary crop breeding is now possible with genome editing (GE) technology based on the prokaryotic CRISPR–Cas9 system [7,13,14].

GE provides refinement of earlier GM technologies. One GE capability is to add, remove, or change DNA sequences without introducing DNA from other species, via the -SDN 1 procedure [5,15]. Targeted base pair changes at the gene level are indistinguishable from those arising with induced mutations, something breeders have used with no ill effects for over a century. 

GE can also involve larger DNA changes and transfers with the SDN 2 and SDN 3 procedures [9,11,15].

Any relaxation or changes of regulations for gene technologies is strongly opposed by the ‘Green’ lobby. These regulations are based on the transfer of DNA from another species and apply the process driven by the ‘precautionary principle’ rather than the ‘outcome’ benefits of the GM product [4,9,14]. Additional costs to market range from US $20–136 million for each GM trait or event in comparison to conventionally bred crops [5,9,15].

With over 20 years of experience, the health risks associated with GM food are clearly no greater than for non-GM foods [5,9,16,17]. Potentially, there are large health benefits and large environmental benefits rather than risks [17,18]. The purpose of this paper is to advocate an equal playing field for the breeding of conventional and GM/GE crops.

This paper addresses GM crops globally, regulation, genome editing (GE), trade, the anti-GM ‘Green’ campaign, food labelling, public education, Australian GM crops and regulations, and a revised National Gene Technology Scheme (NGTS) proposed for food and fiber crops only.

## 2. Review—GM Crops Globally

### 2.1. GM History

Over 20 GM crop commodities had been commercialized by 2018, led by soybeans, with a 78% share of world production; maize: 31%; cotton: 76%; canola: 29% [2]. In addition, GM crops of potato, apple and alfalfa (lucerne) are cultivated in North America, papaya in Hawaii, and eggplant (brinjal), safflower, pineapple and sugar cane in different countries. Leading countries for GM crops in 2018 were USA (75 million ha), Brazil (51), Argentina (23.9), Canada (12.7) and India (11.6), in addition to Paraguay, China, Pakistan, South Africa and Australia, with 0.8–5 million ha [2,19]. Thus, a diversity of GM crops has been adopted worldwide since the first plantings in the mid-1990s. In USA, over 90% of GM soybean and maize are used for stock feed and ethanol production. They also provide highly processed ingredients in foods. Direct GM food consumption includes papaya, sweet corn, squash [11], potato, apple and eggplant.

The global GM seed market was $20.1 billion in 2018 (about $100/ha of GM) and is projected to reach $30.2 billion by 2026 [20]. Companies that develop GM crops must recoup the very considerable costs of compliance with GM regulations, administration, and multi-agency approvals. After the initial GM crop in 1996, the next decade was dominated by development by large agricultural chemical companies (Bayer, Monsanto, Dow, Du Pont and Syngenta), which also sold associated herbicides. There are now additional developers of GM crops in many countries—notably India, China and Bangladesh—with a wider range of GM traits [20].

### 2.2. GM Regulation

GM regulation in many countries is based on the ‘precautionary principle’; all GM crop development must undergo detailed case-by-case assessment of risks to food safety and to the environment, with research and development often conducted in contained facilities. This is expensive research [21,22]. The science of gene technology is poorly understood publicly, enabling the Green lobby to demonize GM for socio-economic reasons, to challenge details of scientific studies [23], and to raise fears that exemption from regulation of SDN 1 GE (no DNA transfer) would allow ‘GM’ foods (defined by the Greens as any DNA change) to be un-labelled and hidden from the public [24,25]. Such objections are not scientifically justified.

Foods derived from GM crops pose no greater safety risk than conventional plant breeding [5,6,19,26]. GM food safety has been validated by over 25 years of research by the American Medical Association and the World Health Organization [10,27,28], in addition to 500+ independent institutions. GM crops benefit the environment by substantially reducing the use of toxic pesticides and herbicides [18].

### 2.3. Genome Editing

GE techniques such as CRISPR enable precise changes to the genome, with cutting of DNA at a specific location as well as the insertion, deletion or modification of nucleotides in a gene; this includes gene silencing, gene enhancement and synthetic genes [5,13,29]. The CRISPR SDN 1 application, which does not involve transfer of DNA from outside the same taxonomic class, is now excluded from biotechnology regulation in USA, Japan and Argentina [22,30]. China has heavily invested in GE with the purchase of Syngenta [31]. GE has been developed for tomato, potato, maize, rice, wheat, sorghum and citrus and presents a major challenge to GM crop regulators [5]. GE dramatically increases the number of traits that can be modified in crops, in a manner far quicker and cheaper than the original GM technology has been able to achieve [29,32]. SDN 1 base pair changes (indels, SNPs, and base substitutions) may be indistinguishable from either a random mutation or what can be achieved by conventional breeding and are regarded as very low risk for human health and the environment [9,33]. They are unlikely, however, to replace existing uses of GM, which pre-date GE technology and are already in farmers’ fields.

The SDN 2 CRISPR procedure involves larger DNA changes with a DNA repair template, while SDN 3 enables precision targeted insertion of foreign DNA; in general, both are subject to GM regulation [6,7]. Occurrences of ‘off-target’ changes are very rare in plants and detectable by whole genome sequencing [9]. These levels of GE provide the ability for insertion of genes from crop wild relatives (CWR), which often have genetic and cytological barriers to crossing with domestic crops. Revised Canadian regulations require novelty to be demonstrated for allowable GE [14,15].

The European Union (EU) and New Zealand continue to classify all GE applications as subject to existing GM legislation, which prohibits all GM crops. Only in Spain and Portugal are commercial cultivation allowed [7,8,9]. However SDN 1 GE does raise challenges in the EU; enforcement is difficult since these changes are indistinguishable from mutagenic changes, which are exempt from EU GM regulation [22].

Future challenges include a warmer, more variable climate, for which CWR can provide genes for abiotic and biotic stress tolerance. In many cases, GE can facilitate introgression of these stress-tolerant traits from CWR into crops [5,34]. These would provide a springboard to address climate change challenges to agriculture production and food security for a fast growing world population. In contrast to the framing of GM regulation, established around 2000 to address fears concerning the manipulation of DNA, there is now an imperative to widen the genetic diversity available for crop breeding to facilitate the sustainability of food security for the future in conjunction with improved agronomy [9].

### 2.4. GM Regulation and Genome Editing

Policies on GM regulation are evolving with changes in biotechnology but at different rates and to different extents in various countries. Precision GE, with little or no downside risk, enables alteration of regulatory policy to focus on ‘outcomes’ or the benefits of traits introduced (as in Argentina [24,32]) in contrast to the traditional ‘process-triggered’ GM regulatory system championed by Europeans [6,7,8,9]. The EU does not exempt any level of GE from GM regulations [5,8,33]. Mutation breeding has always been exempt from regulations, a precedent for relaxing regulations for GE [9,22].

### 2.5. Trade

International trade is affected by national differences in GM regulation and patenting. Imports of GM food may be refused or even banned [8,35]. Usually there are lesser restrictions on importing GM feed products derived from maize and soybeans. Regulations also cover levels of adventitious contamination that are tolerated in non-GM crop imports [35,36]. Contamination is difficult to avoid since the supply chain for transport and storage of crops may not be wholly segregated, resulting in trace quantities of GM in non-GM produce; however, hypersensitive testing for GM presence to 0.00001% level by weight is now possible. Generally, 0.9% is accepted for adventitious GM presence [37]; however, this can vary internationally. Sometimes marketers may not obtain patent authorization for new GM crops for all export destinations, or approvals may be delayed in some areas. These asynchronous approvals result in difficulties with product delivery. Thus, there are significant trade barriers for GM crops, especially with the rapid increase in the number of GM crops; thus, there is a need for trade consensus for GM international commercialism [22]. Needless to say, many NGOs lobby vigorously against giving approval for growing GM crops and importing GM products into the EU and developing countries [36].

## 3. Review—Anti-GM Lobbies

### 3.1. History of Anti-GM

The scientists who developed the first GM traits 30 years ago were cautious with this new technology and considered regulation. However, they had little to do with the current widespread legislation to constrain GM crops with regulations and licences; actions were driven by unsubstantiated claims, fear and uncertainty about GM technology [11,38]. 

The Green campaign was based on emotionalism about dangers to the environment and fear of the health consequences of ‘Frankenstein’ GM food. “These fears spread like wildfire, and within a few years GM was essentially banned in Europe, and such ideas were exported by NGOs like Greenpeace and Friends of the Earth to Africa, India and the rest of Asia, where GM is still banned today” [39]. However, after reviewing the scientific literature, Mark Lynas, a former anti-GM Green leader who destroyed GM field trials in the UK and Australia, admitted that this campaign lacked a scientific basis, that GM food was safe, and that GM crops could benefit the environment. He remarked that “Biotechnology has not been stopped, but regulation has made it prohibitively expensive to all but the very biggest corporations” [39]. 

However, the Green anti-GM lobby still attracts significant funding, with tax-deductible donations of $1 trillion over 2012–2016 in the USA, from wealthy foundations, who donate to lobby groups such as Greenpeace, Organic Consumers Association, Sierra Club and Friends of the Earth [40]. Well-funded campaigns of misinformation on biotechnology, with non-scientific health and environmental claims, support risk regulation of GM crops and discourage developing countries from approving GM crops [24,25,36,40]. Organic certification demands no GM products, so the organic industry has a large vested interest in denigrating GM.

### 3.2. GM Labelling

Green lobbies have argued the need for a free and informed public choice about the presence of GM-derived food ingredients in processed food [11,41,42]. Whether labelling of food with GM derived ingredients should be mandatory has been very contentious in the USA [27]. Since most maize and soybeans produced in the USA are GM, they are often mixed with non-GM products in the transport supply chain or in processing. Thus, food may contain GM DNA or protein in excess of the threshold of 1% (0.9% in EU) and require GM labelling. This is an additional administrative and financial burden on GM food [11].

Exclusion of GE SDN 1 procedure from GM regulation in the USA [5,9,28], which is also proposed for the UK [33,41], is resisted by the Green lobby, as GM food labelling would not be needed for these GE products and the public would not be informed [42]. 

Public opinion worldwide is in favor of transparent labelling with notification of GM presence in food [11,24,43,44,45,46]. Some surveys show that in contrast to scientists a majority of the public (81% in the USA, 84% in China) are concerned about the safety of GM food [11,46], though other concerns such as hormones or chemical pollutants are also relevant [42,47,48]. The Green lobby has been successful in sowing seeds of doubt about GM food and has supported organic food, free from manufactured chemicals and free of GM. The public has a right to be informed about food but often lacks information about GM food [5,11,28,45,47,48]. The gap between the public and scientific views clearly demonstrates the need for public education on biotechnology.

### 3.3. Public Education

A range of groups provide information on biotechnologies supporting their sectoral viewpoints; however, this can cause confusion in the community on the pros and cons of biotechnologies [24,42,49,50]. Better communication is needed on all aspects of biotechnologies so that the public can be well informed. Existing attitudes and perceptions will need to be acknowledged to enable informed choices based on evidence and policies based on fact and not fear [11].

A public awareness campaign is needed on the benefits and safety of crop biotechnology and on the wide variety of biotechnology tools now available, in order to counter well-funded ‘Green’ campaigns [41,42,50,51]. Easy-to-read guides have been produced by ABCA and AAS [5,13], and high-school-level descriptions are now available [52]. Education on GM food safety is needed to prepare students and the public for a world with new biotechnology [51]. This is recognized in China, with a call for scientists to communicate on social media platforms such as WeChat and Weibo [47]. 

This is a contest against the Greens for the hearts and mind of the public, and a concerted information campaign will need to be championed by governments, scientists, industry leaders, educators and on social media [51]. However public acceptance also requires a change in values, justified by an unprecedented surge in world population that exacerbates the challenge of climate change to food security [53,54]. 

## 4. Climate Change and Genetic Adaptation

World food security has become severely threatened since the introduction of regulations on gene technology for crops over 20 years ago. The world situation has changed adversely, with more variable and challenging crop environments; this needs to be recognized in gene technology regulation.

There has been an unprecedented growth in world population by over three-fold in the last 100 years, to 7.85 billion today. This is associated with an equally dramatic 60% rise in the greenhouse gas CO_2_, from additional consumption of coal, oil, gas and cement especially, to over 400 ppm [53,54], resulting in a fluctuating increase in global mean temperature towards 1.5 °C above pre-industrial levels since 1900 [55]. In most scenarios, this warming will rise above 2 °C by 2100, with the lowest emission scenario very unlikely to eventuate due to increasing urbanization and more energy-intensive lifestyles. Certain trends, such as polar warming, can set up reinforcing feedback loops for warming: ice melts, permafrost thaws, and desertification results. Spikes in high temperature will be from a higher base, and frosts and droughts will be more severe, especially upon seed set. Food security will be under threat [34,56].

Thus, the impact of the climate crisis on agriculture has intensified since the 1990s, when genetic modification of food and fiber crops raised safety concerns. However GM crops have been shown to be beneficial, with improvements in crop and food nutrition, disease and pest resistance, yield productivity, and tolerance of drought, high temperature, frost and salinity.

Now, in the 2020s, there is an urgent need to widen the genetic diversity of food and fiber crops to address the coming challenges of abiotic and biotic crop stresses due to climate change [34,56]. GE provides the tools to exploit the largely untapped genetic diversity of CWR, the evolutionary ancestors of crops [56,57], with precise introgression of genes for abiotic/biotic tolerances. CWR have genetic diversity for adaptation to far more extreme environments than crops were exposed to during domestication over the past 12,000 years; they provide opportunities to transform crop adaptation to address climate change [58,59,60].

Agriculture has the task of feeding over 9 billion people by 2050 [56,57], which will rise further towards 2100; however, production environments will become more adverse under climate change. The application of GE and its continuing innovations to address new adaptation requirements of crops needs to be facilitated and promoted rather than hindered by outdated regulations. Continued crop improvement will be part of the manifold solutions for adjusting agriculture to climate change. Failure to update GM regulations will expose the future world to more extensive health and environmental hazards in terms of food security than the perceived risks inherent in the ‘precautionary principle’ [57].

## 5. Review—GM Crops in Australia

### 5.1. GM History in Australia

Australia had 714,000 ha of GM crops in 2015, comprised of herbicide-tolerant canola (444,000 ha), stacked GM (herbicide-tolerant plus pest resistant) cotton (253,000 ha) and herbicide-tolerant-only cotton (20,000 ha) [3,10]. 

The adoption of GM cotton has substantially reduced pesticide use, with benefits to human safety, adjacent livestock enterprises, and the environment as well as improved yields [9,10,61]. Herbicide-resistant canola provided weed control for canola crops and raised yields and profits [19,62]. Crop herbicide resistance provides a key alternative to cultivation for pre-sowing and in-crop weed control. These GM crops can be grown with minimum tillage, thereby conserving soil moisture for crop maturation in the low rainfall southern cropping zone, where every mm saved results in 20 kg/ha or more grain [63]. Herbicide weed control allows earlier sowing to better match crop growth with seasonal winter rainfall. 

A moratorium on GM crops in South Australia (SA) was lifted in 2020, based on the report of Anderson [64]. SA was the last mainland state to have a moratorium, scheduled to last until 2025. The moratorium was estimated to have cost $33 million over 2004–2018, and an additional $5 million was projected for the period up to 2025. GM canola, with a 10% yield benefit, suffered no international market disadvantage compared with non-GM canola; the exception to this was Japan, where an estimated price premium of $32/tonne (about 7%) was paid for GM-free (zero adventitious contamination) canola from Kangaroo Island (KI) in SA under a special market agreement [64]. This entailed segregation of non-GM from GM canola in the delivery chain, with identity protocols and codes of practice. The moratorium was kept for KI crops, and the market chain for KI produce will remain segregated; local councils could appeal before November 2020.

GM crops have been banned in Tasmania since 2001 [65]. Horticulture and honey producers are in favor of this ban to maintain Tasmania’s image for pure GM-free produce.

### 5.2. Regulation of GM Crops in Australia

The National Gene Technology Scheme (NGTS) in Australia dates from the Gene Technology Act of 2000. This enabled regulation, administered by the Office of the Gene Technology Regulator (OGTR), to apply a process-based ‘precautionary’ approach to any kind of directed genetic alteration to a genetically modified organism (GMO) [4]. 

The object of the Act for all living organisms is ”To protect the health and safety of people, and to protect the environment, by identifying risks posed by gene technology, and by managing those risks through regulating ‘dealings’ with GMOs”. 

The release of GM products is strictly controlled by OGTR in coordination with other agencies, including Food Safety Australia and New Zealand (FSANZ), the Australian Pesticides and Veterinary Medicines Authority (APVMA), Therapeutic Goods Administration (TGA), the National Industrial Chemical Notification and Assessment Scheme, the Department of Agriculture and Water Resources, and the Department of the Environment and Energy [4,38]. 

NGTS [4] recognized that CRISPR and other genome editing tools are able to alter genetic expression without the transfer of new genetic material. Therefore OGTR has made an incremental change to a ‘principles based’ flexible approach, with recognition of GE without DNA introduction (SDN 1) and a product history of low risk [4,5,38]. However, OGTR risk assessment and oversight remain in addition to the regulations of complementary agencies.

GM regulations for crops with SDN 1 GE are partially relaxed under the classification ‘Notifiable Low Risk Dealings’ (NLRD) [4,14,38,66]. NLRD reduces the level of compliance documentation but still requires development to be in contained certified facilities and not released to the environment without approval, compliant with OGTR regulations for transport, storage and disposal, and GM field trials that are registered and isolated [4]. NLRD status but must be approved by the Institutional Biosafety Committee (IBC) and OGTR [4,38]. A ‘principles based’ approach leaves in place many of the administrative barriers and costs for risk assessment and management. NLRD for GE remains based on the ‘precautionary’ principle, rather than being ‘outcome’ based, with benefits to society and the environment [14,66]. 

If a perceived risk to the environment still remains after approval by FSANZ for product consumption, objection should be scientifically based rather than emotional. It needs to be noted that CWR populations will alter in response to climate change and are already changing [67]. Whether or not a release of novel GE traits into the environment results in a genetic change to CWR will depend on its selective advantage under climate change. 

There is a future opportunity cost in not recognizing that climate change combined with an unprecedented growth in population creates an urgency to re-adjust GM regulation to promote and accept new gene technologies, especially GE. NGTS can re-align towards an aspiration of crop adaptation (climate proofing) to climate change. Advances in cropping ingenuity and crop genetics will be essential to produce more food in more hostile environments.

## 6. Conclusions

### 6.1. Proposal for a Revised NGTS for Food and Fiber Crops Only—Australia

An appropriate tiering of regulation for crops should recognize outcomes of product benefits to farming and the environment as well as a long-established food safety record.

Arguably GM, and now GE, food and fiber crops should be exempt from NGTS regulation. A revision of NGTS is proposed by the Victorian branch of Agriculture Institute of Australia to facilitate GE for improved productivity growth and tolerance of abiotic and biotic stresses (Redden *pers comm.*). The current NGTS/OGTR over-regulation stifles R&D, raises costs, and tends to exclude GM/GE research and development from small research organizations. The present costs to market for GM/GE crops are prohibitive. The current NGTS/OGTR as applied to crops is no longer fit for purpose; NGTS could be restructured to exempt food and fiber crops but still retain regulations for other GE applications such as vaccine and pharmaceutical crops, micro-organisms and animals [14]. A revision of the NGTS goal is needed from applying the ‘precautionary principle’ for managing risk to proactively encouraging transformative genetic biotechnology for food and fiber crop improvement. 

A Revised NGTS for food and fiber crops would have a new aim: genetic improvement of food and fiber crops by application of gene technologies, with recognition of product outcomes with agricultural, health and environmental benefits. 

This Revised NGTS would greatly reduce operational costs of the plant-centric OGTR and better secure its funding sustainability by removing the monitoring, surveillance and compliance activities for GM/GE food and fiber crops. It would include existing and new biotechnologies. The absence of exorbitant regulation and compliance costs encourages new market entrants for crop biotechnology.

All states and territories would be involved through the Legislative & Governance Forum for Gene Technology (LGFGT), supported by the Gene Technology Standing Committee. Issues of a moratorium, GM and non-GM crop segregation and adventitious contamination would remain the purview of states and territories.

Notification of GM crops with the restructured OGTR may assist with international trade requirements and commitments [34], although importers would still need to update and modify their regulations [22].

### 6.2. New Role for OGTR with Food and Fiber Crops

Consistent with a new NGTS role, a restructured OGTR could change from merely regulating GM food and fiber crops to also educating the public on new biotechnologies, GM food and feed safety and benefits to the environment, using publications, educational webinars and social media posts. OGTR is conversant with GE and has the required expertise to explain and illustrate new developments in biotechnology [4,38,66]. This campaign could be backed up with championing the importance of a Revised NGTS for food security via GE in a more populous world with a changing climate [53,54,55].

## 7. Summary of a Proposed Revision of NGTS for Crops in Australia

OGTR regulations on GM food and fiber crops need to be removed for equivalence with conventionally bred crops. Recognizing that there are community concerns with the use of biotechnologies for crop improvement, the Revised NGTS includes the following:

- Regulatory transparency—The regulations of relevant agencies, such as OGTR, FSANZ and APVMA, should be science-based and supportive of GM products; they will need harmonization between states and in the Commonwealth through the LGFGT Forum, in which all ministers are represented.
- Revision of NGTS/OGTR legislation to exempt GM food and fiber crops from regulation, licensing, surveillance, research containment, transport restrictions, and registering of field trials—Food from both conventionally bred and GM crops would still need to comply with FSANZ standards.
- Climate change challenges for world food security—All food and fiber crops will need all available gene technology tools to be supported and deployed to meet the challenge of feeding the world, even as environments become less hospitable for crop production.
- Research Development and Extension (RD&E)—Research organizations should commit funding for RD&E in crop biotechnology for improved productivity, food nutrition, and adaptation to abiotic and biotic stresses (heat, frost, and drought tolerances, salinity, and pest and disease resistance) for tolerance of climate change via introgression from CWR. Notably, the realizing of benefits from CWR for adaptation to climate change will be considerably enhanced by exemption of GE at all levels from OGTR regulation. Current regulations stifle this opportunity.
- Education—Public understanding of gene technologies is very poor. An education campaign is needed across government policy and social media, with education from primary to tertiary levels. A restructured OGTR could champion public education on the history and benefits of crop genetic improvement, from conventional breeding to advanced biotechnology. This could be reinforced with an emphasis on the food needs of a growing population and the risks to crop production posed by climate change.
- Scientifically based objections—Risk objections to GM crops and derived foods should be science-based, take into account medical expertise on health risk, and consider the various social and environmental benefits.
- Broadened scope—Relaxation of regulations for crop GE would facilitate new market entrants for GM crops beyond the current dominance by multinational companies and would broaden the scope of GE across more crops and key traits.
- Labelling—Individuals may wish to choose foods according to whether they are GM-derived or from conventional crops. Labelling requirements would need to be realistic and not place unnecessarily onerous conditions on producers of GM-derived foods.
- Trade—GM produce should not be disadvantaged vis-à-vis other participants in export or import markets by the application of differing state restrictions. These impose unfair barriers on both internal and external trade, which is contrary to the historic free-trade position of Australian governments.
- Co-existence of GM and non-GM crops—The development of segregation protocols for GM and organic products and of stack management practices at grain receival points show that dual systems are manageable. Individuals or regions wishing to produce for niche markets can do so through protocols between seller and buyer, which already exist for the organics industry.
- Organic food—Genetic modification also benefits the organics industry. Genetic resistance to pests and diseases obviate the use of pesticides and insecticides; GE used for tolerance of abiotic stresses can improve adaptation to a changing climate for vegetable and other minor crops.

Not included in this Revised NGTS is the development of GM crops and micro-organisms for vaccines and pharmaceutical purposes for both animals and humans, for which the current OGTR regulations should remain.

The Revised NGTS proposal is for an exemption of GM food and fiber crops from current NGTS regulation for the continued improvement and sustainability of agriculture. 

## Data Availability

Not applicable.

## References

[1] Parisi C., Tillie P., Rodríguez-Cerezo E. (2016). The global pipeline of GM crops out to 2020. Nat. Biotechnol..

[2] ISAAA (2018). Global Status of Commercialized Biotech/GM Crops.

[3] (2019). Information on genetically modified organisms. Government of Western Australia, Food and Agriculture Newsletter.

[4] (2018). The Third Review of the National Gene Technology Scheme.

[5] (2020). The Official Australian Reference Quide to Agricultural Biotechnology and GM Crops.

[6] Friedrichs S., Takasu Y., Kearns P., Dagallier B., Oshima R., Schofield J., Moreddu C. (2019). An overview of regulatory approaches to genome editing in agriculture. Biotechnol. Res. Innov..

[7] Schmidt S.M., Melinda Belisle M., Frommer W.B. (2020). The evolving landscape around genome editing in agriculture Many countries have exempted or move to exempt forms of genome editing from GMO regulation of crop plants. EMBO Rep..

[8] Kearns P.W.E., Kleter G.A., Bergmans H.E.N., Kuiper H.A. (2021). Biotechnology and Biosafety Policy at OECD: Future Trends. Trends Biotechnol..

[9] Turnbull C., Lillemo M., Hvoslef-Eide T.A.K. (2021). Global Regulation of Genetically Modified Crops Amid the Gene Edited Crop Boom—A Review. Front. Plant Sci..

[10] Brookes G. (2016). Adoption and impact of genetically modified (GM) crops in Australia: 20 year’s experience. PG Economics Report.

[11] Jaffe G. (2016). Genetically Engineered Foods and their Regulation: The Way Forward after Twenty Years of Adoption. Regulatory Focus.

[12] The Royal Society (2018). Genetically Modified (GM) Plants: Questions and Answers. https://royalsociety.org/topics-policy/projects/gm-plants.

[13] (2019). Genetic Modification-Questions and Answers.

[14] (2021). Modernising and future-proofing the National Gene Technology Scheme: Proposed regulatory framework to support implementation of the Third Review of the Scheme. Consultation Regulation Impact Statement.

[15] Coghlan A. (2014). Relaxing of Rules Urged for Genetically Modified Crops. https://www.newscientist.com/article/dn25222-relaxing-of-rules-urged-for-genetically-modified-crops/#ixzz6Uy5EaITQ.

[16] Delaney B. (2015). Safety assessment of foods from genetically modified crops in countries with developing economies. Food Chem. Toxicol..

[17] (2019). Safety of Genetically Modified (GM) Foods. Government of Western Australia, Food and Agriculture Newsletter.

[18] Brookes G., Barfoot P. (2016). GM Crops: Global Socio-Economic and Environmental Impacts 1996–2016.

[19] Brookes G. (2020). Crop Biotechnology Continues to Provide Higher Farmer Income and Significant Environmental Benefits.

[20] Fortune Business Insights (2019). Genetically modified seed market size, share and industry analyses by crop (corn, soybean, cotton, canola and others) and regional forecast 2019–2026. Agriculture-Genetically Modified Seed Market.

[21] Nicolia A., Manzo A., Veronesi F., Rosellini D. (2013). An overview of the last 10 years of genetically engineered crop safety research. Crit. Rev. Biotechnol..

[22] Menz J., Modrzejewski M., Frank Hartung F., Wilhelm R., Sprink T. (2020). Genome Edited Crops Touch the Market: A View on the Global Development and Regulatory Environment. Front. Plant Sci..

[23] Gennaro A. European Food Safety Authority Rebuffs Activist Criticism of Its Safety Assessment of Insect and Herbicide Resistant GMO Crops. European Food Safety Authority 8 July 2020. Genetic Literacy Project. https://geneticliteracyproject.org/2020/07/08/european-food-safety-authority-rebuffs-activist-criticism-of-its-safety-assessment-of-insect-and-herbicide-resistant-gmo-crops.

[24] Clemons R., Potter A. (2016). Are You Eating Genetically Modified Food.

[25] Druker S. (2014). The UK’s Royal Society: A Case Study in How Health Risks of GMO’s Have Been Systematically Misrepresented.

[26] McDonald R.S. (2014). Safety of Genetically Modifies Food and Organisms.

[27] (2012). American Medical Association: GMO Labelling Not Necessary.

[28] (2016). Frequently Asked Questions on Genetically Modified Foods.

[29] Zhang Y., Massel K., Godwin I.D., Gao C. (2018). Applications and potential of genome editing in crop improvement. Genome Biol..

[30] Stokstad E. (2020). United States relaxes rules for biotech crops. Science and Policy.

[31] Cohen J. (2019). To Feed 1.4 Billion, China Gets Big on Genome Editing of Crops. https://www.sciencemag.org/author/jon-cohen.

[32] Godwin I. Gene Editing Opens a Faster and Cheaper Way to Introduce New Crop Traits.

[33] Lyon N. (2020). Gene editing opens faster, cheaper way to introduce new crop traits. Grain Central News.

[34] Coyne C.J., Kumar S., Wettberg E.J.B., Marques E., Jens D., Berger J.D., Redden R.J., Ellis N.T.H., Jan Brus J., Zablatzká L. (2020). Potential and limits of exploitation of crop wild relatives for pea, lentil and chickpea improvement. Legume Sci..

[35] Frisvold G., Reeves J. (2015). Genetically modified crops: International trade and trade policy effects. Int. J. Food Agric. Econ..

[36] Smyth S.J. (2017). Genetical modified crops, regulatory delays and international trade. J. Food Energy Secur..

[37] Betzner A. (2016). National Limits Set for GM Canola Presence.

[38] OGTR (Office of Gene Technology Regulation) (2020). Technical Review of the Gene Technology Regulations 2001. Discussion Paper: Options for Regulating New Technologies.

[39] Lynas M. (2013). Lecture to Oxfords Farming Conference. Environmental News and Comment.

[40] Genetic Literacy Project (2020). Anti-GMO Advocacy Funding Tracker: Vast network of donors and NGOs seed doubt about crop biotechnology. Newsletter GLP Team.

[41] Gusovsky D. (2014). Big lobbying dollars flow in engineered food fight. Agriculture.

[42] GM Watch (2019). Center for Science in the Public Interest, Greg Jaffe, Cornell and GMOs. https://www.gmwatch.org/en/news/latest-news/18687-center-for-science-in-the-public-interest-greg-jaffe-cornell-and-gmos.

[43] CropLife (2018). Submission to Consultation Paper on Food Derived Using New Breeding Techniques.

[44] Mackelprang R. (2018). Organic Farming with Gene Editing: An Oxymoron or a Tool for Sustainable Agriculture?.

[45] Miles S., Ueland O., Frewer L.J. (2005). Public attitudes towards genetically-modified food. Br. Food J..

[46] Cui K., Shoemaker S.P. (2018). Public perception of genetically-modified (GM) food: A Nationwide Chinese Consumer Study. NPJ Sci. Food.

[47] Wang Q. (2015). China’s Scientists Must Engage the Public on GM. Nature 591. https://www.nature.com/news/china-s-scientists-must-engage-the-public-on-gm-1.17031.

[48] EU (European Commission) (2010). European Research Area. A Decade of EU-Funded GMO Research (2001–2010). Project Information. https://ec.europa.eu/research/biosociety/pdf/a_decade_of_eu-funded_gmo_research.pdf.

[49] (2000). Safety Aspects of Genetically Modified Foods of Plant Origin: Report of a Joint FAO/WHO Expert Consultation on Foods Derived from Biotechnology.

[50] Freedman D.H. (2013). Are Engineered Foods Evil?. Sci. Am..

[51] Dewey C. The Government Is Going to Counter ‘Misinformation’ About GMO Foods. Washington Post, 4 May 2017. https://www.washingtonpost.com/news/wonk/wp/2017/05/03/the-government-is-going-to-try-to-convince-you-to-like-gmo-foods.

[52] Cavaliero L. (2020). Year 10 Biology, Genetic Modification. Library and Innovation Centre. https://library.plc.wa.edu.au/year10/biology/GM/genetic_modification.

[53] Our World in Data (2019). CO_2_ Emissions by Fuel Type, World. Global Carbon Project: CDIAC. https://ourworldindata.org/co2-and-other-greenhouse-gas-emissions.

[54] Our World in Data (2019). Atmospheric CO_2_ Concentrations. EPICA Dome C CO_2_ Record (2015) & NOAA (2018). https://ourworldindata.org/co2-and-other-greenhouse-gas-emissions%20%E2%80%A2%20CC%20BY.

[55] United States Environmental Protection Agency (2017). Future of Climate Change. Climate Change Science.

[56] Zhang H., Mittal N., Leamy L.J., Barazani O., Song B.H. (2017). Crops: Back into the wild—Apply untapped genetic diversity of wild relatives for crop improvement. Evol. Appl..

[57] Scheben A., Edwards D. (2017). Genome editors take on crops. Science.

[58] Hajjar R.A.E., Hodgkin T. (2007). Use CWR in major crops for key traits 1997–2007. The use of wild relatives in crop improvement: A survey of developments over the last 20 years. Euphytica.

[59] Djanaguiraman M., Vara Prasad P.V., Jackson M., Floyd-Lloyd B., Band Parry M. (2014). Crop wild Relatives, trait tolerance to High Temperature Stress, Ch12. Plant Genetic Resources and Climate Change.

[60] Xue G.P., McIntyre L., Yadav R.H., Lotze-Campen H. (2011). Wild relative and transgenic innovation for enhancing crop adaptation to warmer and drier climate. Ch11. Crop Adaptation to Climate Change.

[61] Naranjo S.D., Ellsworth P.C. (2009). Fifty years of the integrated control concept: Moving the model and implementation forward in Arizona. Pest. Manag. Sci..

[62] ACIL Tasman (2007). GM Canola: An Information Package.

[63] Sadras V.O., Angus J.F. (2007). Benchmarking water-use efficiency of rainfed wheat in dry environments. Field Crops Res..

[64] Andserson K. (2019). Independent Review of the South Australian GM Food Crop Moratorium. Report to the SA Minister for Primary Industries and Regional Development.

[65] Breen F. (2019). Tasmania’s GMO ban good news for some, a ‘missed opportunity’ for others. Tas Country Hour.

[66] Thygesen P. (2019). Clarifying the regulation of genome editing in Australia: Situation for genetically modified organisms. Trans. Res..

[67] Nevo E., Henry R., Redden Y., Maxted D., Guarino S. (2015). Ch 3. Global warming and evolution of wild cereals. Crop Wild Relatives and Climate Change.

